# φXANES: *In vivo* imaging of metal-protein coordination environments

**DOI:** 10.1038/srep20350

**Published:** 2016-02-10

**Authors:** Simon A. James, Dominic J. Hare, Nicole L. Jenkins, Martin D. de Jonge, Ashley I. Bush, Gawain McColl

**Affiliations:** 1 The Florey Institute of Neuroscience and Mental Health, The University of Melbourne, Parkville, Victoria, 3052, Australia; 2 Elemental Bio-imaging Facility, University of Technology Sydney, Broadway, New South Wales, 2007, Australia; 3 Australian Synchrotron, Clayton, Victoria, 3168, Australia

## Abstract

We have developed an X-ray absorption near edge structure spectroscopy method using fluorescence detection for visualizing *in vivo* coordination environments of metals in biological specimens. This approach, which we term fluorescence imaging XANES (φXANES), allows us to spatially depict metal-protein associations in a native, hydrated state whilst avoiding intrinsic chemical damage from radiation. This method was validated using iron-challenged *Caenorhabditis elegans* to observe marked alterations in redox environment.

Metal cofactors represent a fundamental component of biochemistry *via* their ability to facilitate electron transport and stabilize biomolecules. An important requirement for understanding the role of transition metals in normal biochemistry and disease processes is the determination of their coordination environment^1^,^2^. The synergy of synchrotron-based X-ray fluorescence microscopy (XFM) and X-ray absorption near edge structure (XANES) spectroscopy represents a powerful analytical approach for studying metal biochemistry at the micro-scale. This permits both quantitative mapping of metal distribution and profiling of the native coordination environment without the need for exogenous molecular probes^3^. We combined these two measurement strategies using the same synchrotron beamline to develop an imaging approach we have called fluorescence imaging (‘fi’, or φ for the Greek ‘phi’) φXANES.

XANES has traditionally been used in biology to profile coordination environments in fixed locations (‘point’ XANES)^4^, rather than functioning as a fine resolution imaging technique. In addition to lack of spatial information is the problem of extended exposure to the ionizing X-rays (>10 keV) that can damage the sample by disrupting chemical bonds. Photoreduction of redox metals can occur at doses around 10^7^ Gy^5^,^6^. Exposure of XANES samples can be increased to 10^10^ Gy using cryogenic conditions (−100 °C)^7^, though samples are still susceptible to morphological damage^8^ and the requirement to maintain the specimen at low temperatures during preparation and measurement increases logistical complexity. Ideally, analysis of samples that remain hydrated and at physiological temperature is preferable. Here, we demonstrate the development of non-destructive φXANES imaging at standard laboratory conditions, validated in a *Caenorhabditis elegans* model of disrupted metal metabolism.

To determine the optimal conditions for φXANES (see Supplementary Note), we tested two experimental scenarios to establish the appropriate dose of radiation to which hydrated and anesthetized *C. elegans* could be exposed without inducing morphological changes and to avoid photoreduction of endogenous iron. These were: i) ‘high dose’ φXANES, where elemental maps of high statistical precision were obtained using long dwell and spatial oversampling in two directions with a symmetrical beam profile; and ii) ‘low dose’ φXANES, where a shorter dwell time was used along with a vertically-elongated beam (reducing the X-ray flux density), while undersampling in the vertical direction (for details of the focused beam see Supplementary Figure 1). Together, these measures reduced sampling time and localized beam exposure by a factor of 100. A representative whole-body XFM elemental map of calcium and iron distribution in a separate cryofixed and lyophilized specimen is presented for anatomical reference (Fig. 1a). For high dose φXANES, four hydrated adults were mapped using standard XFM parameters (Fig. 1b), with an anterior region containing iron-rich intestinal cells in one specimen selected for φXANES. Here, the region underwent XANES analysis spanning the iron K-edge incident energies (7100 to 7220 eV), exposing this region to an estimated 5 × 10^8^ Gy, within the radiation dose range previously reported to stimulate photoreduction of iron^6^.

These four individuals were mapped again using standard XFM following φXANES, approximately 5 hours after the initial XFM scan. We performed intensity correlation analysis (ICA)^9^ on a region of interest representative of the pre and post-φXANES scanned area and found that distribution of pixel intensities significantly differed between the two maps (ICA quotient *Q* = 0.006; 0 = no correlation), demonstrating clear sample damage. In parallel, we selected an anatomically equivalent adjacent specimen that received ~10^6^ Gy from the two XFM maps alone which, in contrast, maintained a consistent iron distribution (*Q* = 0.37; 0.5 = perfect correlation).

Low dose φXANES and XFM of a matching sample group were then examined (Fig. 1c), encompassing an additional four adults. These samples were exposed to 4 × 10^6^ Gy, approximately 100-fold less than the high dose method. Iron spatial distribution pre- and post φXANES was maintained (*Q* = 0.41). Although low dose φXANES does sacrifice some spatial detail (5.6 μm^2^ versus 0.64 μm^2^ sampling area), the reduced radiation dose (<2 × 10^8^ Gy) ensures photoreduction of iron^6^ and other metals^10^ is minimized while endogenous spatial distribution is maintained.

To demonstrate the potential of φXANES to profile bioinorganic chemistry *in vivo*, we examined a combined genetic and exogenously challenged model of severe iron dyshomeostasis. *C. elegans* lacking the iron-storage protein ferritin (both genes *ftn-1* and *ftn-2* are ablated *via* mutation; hereafter referred to as ferritin nulls) have increased oxidative load from elevated ferrous iron^11^, and have a shortened lifespan compared to wild type (Supplementary Figure 2). We designed two experimental paradigms, exposing both wild type and ferritin nulls to either basal iron levels *via* normal culturing conditions, or high iron through supplementation of their growth media. Adults were anesthetized and quantitatively mapped by XFM (Fig. 2a). Exposure to high iron increased levels in wild type animals compared to equivalent animals raised under basal conditions (one-way ANOVA with Tukey’s *post hoc* test; *p* < 0.001; Fig. 2b), whilst ferritin nulls raised on basal iron exhibited a decrease in total levels compared to wild type (*p* < 0.001). As ferritin is not involved in iron uptake, the reduced load is consistent with an inability to store iron^12^; ferritin nulls on high iron still displayed increased total body burden (*p* < 0.001).

Each experimental group was mapped *via* φXANES using our optimized low dose parameters, scanning the iron K-edge (Fig. 2c). This range encompasses the characteristic pre-edge (~7115 eV), shoulder (~7124 eV), and crest (~7130 eV) features, arising from 1s → 3d, 1s → 4s and 1s → 4p electronic transitions, respectively. The precise energy of the pre-edge reflects the relative abundance of ferrous [Fe(II)] and ferric [Fe(III)] iron, and shifts to lower energies in the presence of increased Fe(II)^13^. When comparing the pooled XANES spectra (*i.e.* the mean for all pixels) for each measured individual, we observed that the centroid energy for the pre-edge transition in wild type (7114 eV; Fig. 2d) cultured on high iron was unchanged but the reduced intensity was indicative of an increase in the number of octahedral Fe(III) centres^14^, consistent with increased buffering of iron within ferritin, where it is arranged in such coordination geometry^15^. However, ferritin nulls, regardless of iron load, demonstrated a shift to lower centroid energies away from 7114 eV, indicating increased Fe(II). Comparing the relative intensity of the shoulder and crest features of the iron K-edge in each group further confirmed a disruption in the iron coordination environment. First-derivative iron XANES spectra exhibited a significant alteration in the cumulative Fe(III):total iron ratio between wild type and ferritin null groups (one-way ANOVA with Tukey’s *post hoc* test; *p* < 0.001; Supplementary Fig. 3a,b). This effect was independent of iron loading. In addition, we observed increased variability between ferritin nulls compared to wild type (Bartlett’s test for homogeneity of variances χ^2^ = 16.54; *p* < 0.001; Supplementary Fig. 3c), consistent with a homeostatic system in distress.

Potentially hundreds of individual iron-binding proteins contribute to the proteome (the ferroproteome), although even in microbes the precise number remains unclear^16^. These include proteins containing heme moieties, iron-sulfur clusters, ferrihydrite-like crystalline structures (as in ferritin), and multi-dentate ligands arising from specific amino acid conformations^17^. When examining the cumulative φXANES spectra, we are assessing the aggregate distribution of iron-protein coordination complexes in a whole organism. Spatial mapping by φXANES allows for individual tissue or cell types to be objectively assessed for changes to iron coordination in response to specific challenges at the μm scale. We applied principal component analysis (PCA) and *k*-means clustering (CA) as implemented in the Multivariate ANalysis Tool for Spectromicroscopy (MANTiS)^18^ package after tiling φXANES maps to directly compare spatial coordination states in wild type and ferritin nulls raised on high iron. Pixels with similar XANES spectra were assigned to six distinct regions of interest (ROIs), color coded in Fig. 2e as descending Fe(III):total iron. Of these six regions, ROIs 1 and 5 differed in proportions of total iron-containing pixels between genotypes (Supplementary Table 1). There was a systemic shift towards a lower Fe(III):total iron in ferritin nulls compared to wild type. The intestine consists of highly metabolically active cells in *C. elegans*, which in the ferritin null animals demonstrated the largest shift in iron redox balance, with ROI5 essentially replacing the spatial distribution of ROI1 (Fig. 2e). We confirmed that the absence of ferritin mapped to the changes in ROIs 1 and 5 by subtracting the XANES spectra of purified horse spleen ferritin (the ferrihydrite-like iron core of ferritin is a commonality in all species expressing ferritin homologues^12^) from each ROI (ΔXANES; Fig. 2f–h). While ROI1 (absent in ferritin nulls) shared stark similarities with the reference ferritin standard, ROI5 (markedly increased in ferritin nulls) showed XANES spectra that deviated significantly (Wilcoxon signed-rank test; *W* = 734; *p* < 0.05). Finally, the clear splitting in centroid energy of ferritin-absent ROI5 further supports the higher levels of Fe(II) in the ferritin null animals, consistent with the well characterized role of ferritin in buffering reactive ferrous iron as a redox-silenced mineralized Fe(III) species. For comparison, XANES spectra of additional iron-protein ligands (oxidized and reduced heme-containing cytochrome *c*) are shown in Supplementary Figure 4.

In summary, we have demonstrated that φXANES is a powerful method for mapping coordination environments *in vivo*, with no displacement of target elements and measurement dose well below previous studies of biological iron redox status inline with bulk XAS measurements, and without the need for cryogenic sample environment. φXANES in conjunction with PCA-CA is ideal for assessing changing coordination environments in tissue sections, small model organisms (including *C. elegans* and *Drosophila melanogaster*, which has previously been used for point XANES^19^) and cell culture. Although we validated this method using iron coordination, φXANES can be applied to any element to which XFM is sensitive, drugs that elicit a change in cellular redox environment, and longitudinal studies that require real-time assessment of changing coordination conditions in a biological system.

## Methods

Methods and any associated references are available in the online version of this paper.

## Additional Information

**How to cite this article**: James, S. A. *et al*. ϕXANES: *In vivo* imaging of metal-protein coordination environments. *Sci. Rep.* **6**, 20350; doi: 10.1038/srep20350 (2016).

## Supplementary Material

Supplementary Information

## Figures and Tables

**Figure 1:**
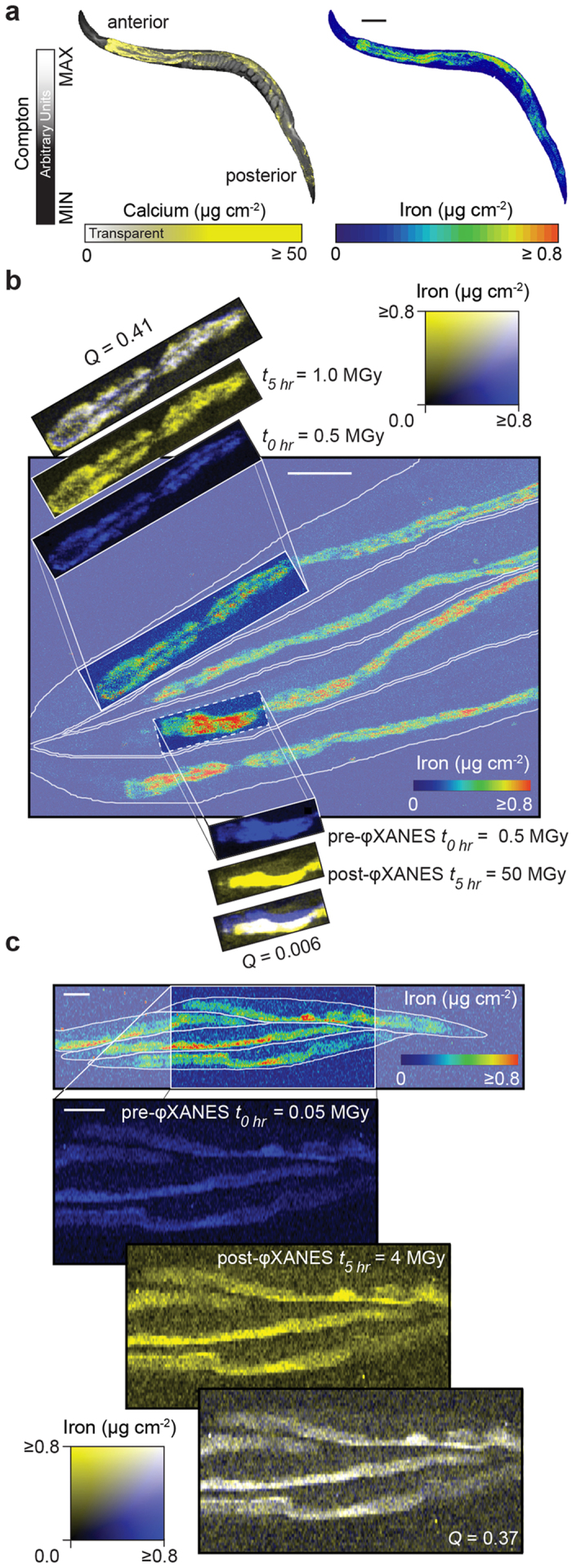
Optimization of φXANES parameters to minimize sample perturbation. **(a)** Reference X-ray fluorescence micrograph showing Compton scatter (greyscale) and quantitation of calcium (yellow) and iron (rainbow color scale) in a dried adult *C. elegans* as a reference of general anatomy. **(b)** A map of iron distribution (rainbow color scale) was recorded at 7282 eV and used to select two sub-regions for reanalysis: the dashed white box was selected for high dose φXANES (114 scans) followed by a final map at 7282 eV, while the ‘control’ area (solid white box) was mapped at 7282 eV twice only; prior to and post completion of φXANES on the first region. With the exception of incident energy, all scan parameters were held constant for this series of measurements and the dose associated with recording each map was ~5 MGy. Comparing the first (blue) and last (yellow) maps (over a 5 hour period, shown in overlay where white represents colocalization) from each sub-region showed that high dose φXANES induced significant redistribution (ICA quotient *Q* = 0.006) of iron compared to the region mapped only twice (*Q* = 0.41). **(c)** The distribution of iron was also mapped using low dose φXANES. Total dose for these maps was 0.05 MGy. ICA comparing the first and last maps revealed strong agreement of iron signal (*Q* = 0.37), consistent with a minimally disturbed system. Scale bar for all images = 100 μm.

**Figure 2:**
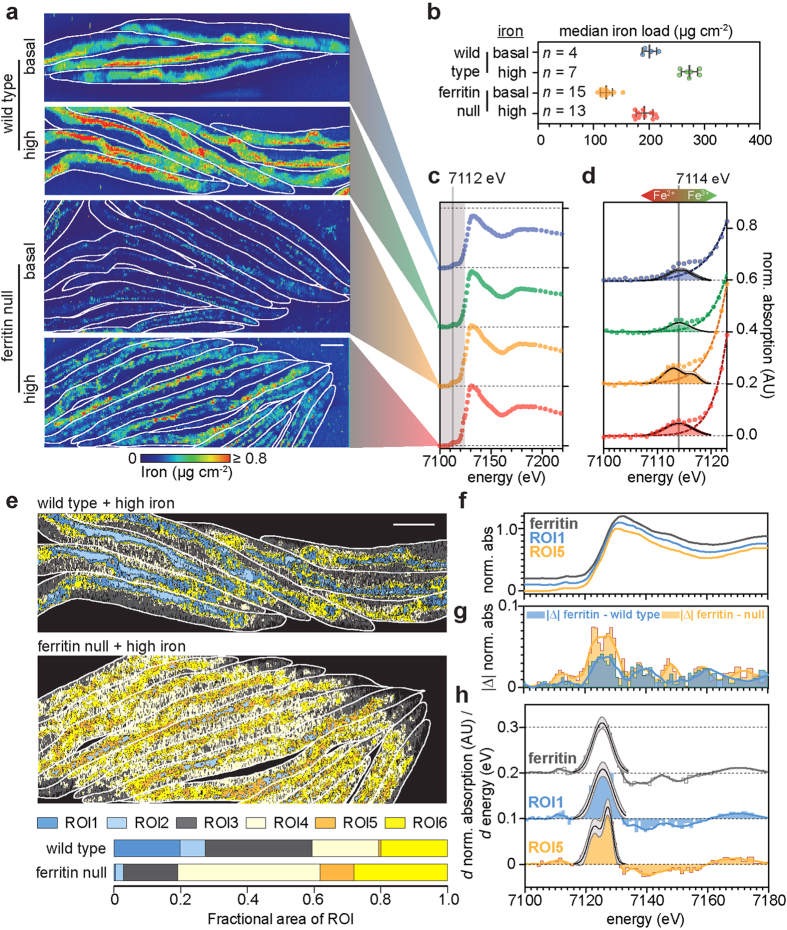
Loss of ferritin skews iron load and Fe(III):total iron ratio. **(a)** XFM of wild type and ferritin nulls ± high iron. Ferritin nulls exhibited reduced total iron, but, as expected, retained capacity to uptake iron *via* a mechanism independent of ferritin. White outline demarcates the boundary of each animal, color table defines iron areal density (μg cm^−2^) and the scale bar = 100 μm. **(b)** Median iron areal density for each specimen, showing elevated iron load following exposure to high iron (*n* = number of specimens per group; data presented as the mean of the medians ± 1 SD). **(c)** Iron XANES spectra (across all pixels) extracted from low dose φXANES for the groups shown in (**a**). The starting position of iron K-edge (7112 eV) is marked with a vertical line and for clarity the integrated XANES spectra from each group has been offset vertically. **(d)** Expanding the pre-edge region (grey box in (**c**)), following subtraction of the rising edge (dashed line), highlights changes in both the energy and intensity of the 1s → 3d pre-edge feature between groups. The extracted data (colored circles) and fitted Gaussian (solid black lines; 95% confidence interval in grey) are superimposed to determine the centroid values (~7114 eV for wild type; marked for reference). Loss of ferritin changed the pre-edge feature to exhibit two centroid energies (7113 eV and 7117 eV), whereas high iron exposure retained a single centroid energy of 7114 eV. **(e)** Areas of similar iron XANES spectra identified via principal component analysis and *k*-means clustering marked as distinct regions of interest (ROIs, six per specimen). The XANES spectra for each cluster were highly structured and allowed the Fe(III):total iron ratio to be calculated for each ROI. The spatial extent of each region as a proportion of the area scanned is shown and highlights that, with the exception of portions of the intestine, the majority of wild type tissues possess relatively low Fe(II) levels despite a higher iron load. In particular, two regions differed significantly in Fe(III):total iron ratio (ROIs 1 and 5; both localized along the intestinal tract) between wild type and ferritin nulls. Scale bar = 100 μm. **(f)** XANES from a purified horse spleen ferritin standard was compared to the cumulative XANES spectra from ROIs 1 and 5. **(g)** The difference (ΔXANES) between these two ROIs and the ferritin standard spectra showed that ROI1 had stark similarities with the ferritin profile, whilst ROI5, which was practically absent in wild types demonstrated significant variation from the ferritin XANES spectra, further supporting complete ablation of ferritin from these animals and an altered coordination environment. **(h)** Features characteristic of electronic transitions used to differentiate between iron oxidation states also revealed that ROI5 had a greater level of abundant Fe(II) compared to ROI1 and the ferritin standard, where the majority of iron is stabilized in a mineralized Fe(III) form.
