# Erratum: φXANES: *In vivo* imaging of metal-protein coordination environments

**DOI:** 10.1038/srep22684

**Published:** 2016-03-21

**Authors:** Simon A. James, Dominic J. Hare, Nicole L. Jenkins, Martin D. de Jonge, Ashley I. Bush, Gawain McColl

Scientific Reports 6: Article number: 2035010.1038/srep20350; published online: 02102016; updated: 03212016

In this Article, the online methods were omitted. The online methods should read:

**Strains**

Wild type *C. elegans* (strain N2) were obtained from the *Caenorhabditis* Genetics Center (University of Minnesota), and *ftn-2*(*ok404*) and the ferritin null *ftn-2*(*ok404*); *ftn-1*(*ok3625*) (strain GMC005) have been previously described^1^. All strains were cultured at 20 °C on standard nematode growth media (NGM) with *E. coli* (strain OP50)^2^. Populations were developmentally synchronized via a 3 hour egg lay and then developed at 20 °C for three days. Cohorts were then transferred to either standard NGM (basal media) or NGM supplemented with 5 mg ml^−1^ ammonium ferric (III) citrate (FAC, C_6_H_8_O_7_·*x*Fe^3+^·*y*NH_3_, Sigma Aldrich; high iron media) for 48 h at 25 °C. The concentration of iron was 246 ng g^−1^ in the basal media and 1.17 mg g^−1^ for high iron media for our batch of reagents^1^.

**Lifespan analysis**

The effects of *ftn-2*(*ok404*) and *ftn-2*(*ok404*); *ftn-1*(*ok3625*) null mutants on lifespan at 25 °C were compared to wild type using protocols previously published^1^.

**Elemental mapping: X-ray fluorescence microscopy (XFM)**

Specimens for analysis were repeatedly washed with s-basal, anesthetized, and mounted on Si_3_N_4_ windows (window area 16 mm^2^, 2 μm thick, Silson, United Kingdom) for analysis at the XFM beamline at the Australian Synchrotron as described previously^1^. In brief, the specimens were cushioned on an agarose pad supported by the Si_3_N_4_ window and overlaid with Ultralene film (4 μm thick, Volga Instruments) to prevent dehydration. Element localization for iron and lighter elements was mapped using an incident beam of 7282 eV X-rays in order to induce K-shell ionisation and to clearly separate the Elastic and Inelastic peaks from the elemental peaks. Dynamic Analysis and deconvolution of fluorescence via GeoPIXE 7.1 (CSIRO) was used to produce quantitative elemental maps^3^. Two single-element thin metal foils of known areal density (manganese 18.9 μg cm^−2^ and iron 52.2 μg cm^−2^, Micromatter, Canada) were used to establish elemental quantification.

X-ray fluorescence (XRF) was corrected for an assumed specimen composition and thickness^4^ using the known composition and thickness of the Si_3_N_4_ window and Ultralene film and composition and density of the agarose. Small deviations from these assumptions are not significant for results presented in this study as the effects of beam attenuation and self-absorption on calcium and iron XRF are negligible for a specimen of this type and size. The sample preparation and elemental mapping of the freeze-dried five-day old (post egg lay) wild type were performed as previously described^4^.

**Fluorescence imaging X-ray absorption near edge structure (φXANES) spectroscopy**

Iron K-edge φXANES was measured as a series of XRF maps (as described above) at a range of incident energies spanning the iron K-edge (7112 eV). Incident energy was calibrated by defining the first derivative peak of the iron foil standard to be 7112.0 eV and the spectrum from this foil was recorded at the beginning and end of the experiment to monitor energy stability. XRF was normalized to the incident beam flux monitored by an ionization chamber with a 27 cm path length placed upstream of the focusing optics and filled with 100% N_2_. The XANES spectra were extracted from pixels within the selected areas and background- and baseline-corrected using methods implemented in ATHENA, an interface to IFEFFIT^5^. The relative intensity of the 1s → 3d, 1s → 4s and 1s → 4p electronic transitions were determined as previously described^1^.

**High dose φXANES**

Specimens were continuously scanned with the resultant XRF binned at 0.8 μm horizontal intervals (Supplementary Fig. 1a). Upon completion of each row the sample was translated 0.8 μm vertically before continuing the raster scan. This process produced elemental maps containing up to 882,096 0.64 μm^2^ pixels with a pixel transit time of 15.6 msec. The full width at half max (FWHM) of a Gaussian distribution fitted to the beam profile defined the spatial extent of illumination as 2.1 μm in the horizontal and 2.8 μm vertical. High dose φXANES imaging was then collected across 114 energies spanning the Fe K-edge (7112 eV) using the following incident energy increments: 7042 eV to 7102 eV in 10 eV steps; 7102 eV to 7110 eV in 1 eV steps; 7110 eV to 7115 eV in 0.5 eV steps; 7115 eV to 7152 eV in 1 eV steps; and 7152 eV to 7256 eV in 2 eV steps. This series of maps delivered a total absorbed dose estimated at 500 MGy. The distribution of iron was mapped at 7282 eV, using identical scan parameters, immediately prior and post high dose φXANES.

**Low dose φXANES**

During data collection specimens were continuously scanned through X-ray focus with the resultant XRF binned at 0.8 μm horizontal intervals. Upon completion of each row the sample was translated 7 μm vertically before continuing the raster scan (Supplementary Fig. 1b). This process produced elemental maps ranging up to 102,000 pixels and containing 5.6 μm^2^ pixels with a pixel transit time of 1.9 msec. As above the beam profile defined the spatial extent of illumination as 1.5 μm in the horizontal and 3.8 μm vertical. Low dose φXANES imaging was collected at 82 energies spanning the Fe K-edge using the following incident energy increments: 7100 eV to 7162 eV in 1 eV steps; 7162 eV to 7192 eV in 2 eV steps; and 7192 eV to 7217 eV in 5 eV steps. This series of maps delivered a total estimated measurement dose of 4 MGy.

**Image analysis**

Analysis of elemental XRF maps, including colocalization calculations of ICA quotients^6^, was performed using a combination of tools native to GeoPIXE and ImageJ, a java- based image-processing program developed by the National Institutes of Health (USA)^6^,^7^. Principal component analysis followed by cluster analysis (PCA-CA) of the φXANES stack was achieved by grouping pixels based on spectral similarity using MANTiS v2.09^8^.

**Statistics**

Lifespan data were compared via a Kaplan–Maier survival curve and non-parametric log rank test. Median iron per individual derived from the iron maps was compared between experimental groups via 1-way ANOVA with Tukey’s *post hoc* tests. Variability of individual Fe(III):total iron ratios derived from φXANES of the various treatment groups was assessed via Bartlett’s test for homogeneity of variances. Non-normally distributed data for each spectra (D’Agostino’s K^2^ test; *p* < 0.05; shown in Fig. 2f–h) required comparing the group medians to an expected value of zero (*i.e.* ΔXANES for ferritin = 0). All tests were performed using Prism v5.0d (GraphPad).
