# Supplementary material for: φXANES: In vivo imaging of metal-protein coordination environments
Source: Sci Rep. 2016 Feb 10;6:20350. doi: 10.1038/srep20350 (PMC4748213; doi:10.1038/srep20350)
Supplement: Supplementary Information [file srep20350-s1.pdf]

Supplementary Information for:

φXANES: *In vivo* imaging of protein-metal coordination environments

Simon A. James[1,3,4], Dominic J. Hare[1,2,4], Nicole L. Jenkins[1], Martin D. de Jonge[3], Ashley I. Bush[1] & Gawain McColl[1]

[1] The Florey Institute of Neuroscience and Mental Health, The University of Melbourne, Parkville, Victoria, 3052, Australia.

[2] Elemental Bio-imaging Facility, University of Technology Sydney, Broadway, New South Wales, 2007, Australia.

[3] Australian Synchrotron, Clayton, Victoria, 3168, Australia.

[4] These authors contributed equally to this work.

Correspondence should be addressed to G.M. (gawain.mccoll@florey.edu.au).

Supplementary note:

Development of hard X-ray fluorescence scanning microscopes has enabled the capture of intricate spatial and chemical details present in complex biological samples. While the push towards ever-finer spatial resolution is driven by the desire to image the finest structures, these details provide the most insight when interpreted within their larger spatial context. The Maia detector system[1,2], used in this study and developed by the Commonwealth Scientific and Industrial Research Organisation (CSIRO, Australia) and Brookhaven National Laboratory (BNL, USA), enables a new approach to X-ray fluorescence microscopy (XFM): where the goal is to maximize information by probing scales of organisation, from tens of millimetres down to a single microns – presenting a challenge to capture detail over ~4 orders of magnitude. Imaging (in two dimensions) over these length-scales requires a capability to produce elemental maps containing $\sim 10^8$ pixels in practical time-scales, *i.e.* < 10 hours. To obtain adequate counting statistics within each of these pixels (*e.g.* $\sim 10^3$ counts) demands a detector system able to process sustained count-rates of around 3×10^6 photons per second. Specifically engineered for the role, the Maia system addresses many critical limitations of other detectors such as slow read-out overheads (leading to excessively long experiments) and poor performance under high-count rates (causing data distortion). The large detector area, positioned in the backscatter geometry, enables the Maia to subtend a solid angle of 1.3 sr at the specimen. The Maia adopts a massively parallel architecture, spreading the high count-rate over an array of 384 individual detector pixels, each of which is capable of a count rate of up to 10 kHz, minimizing the impact of event pileup and degraded energy resolution. The Maia was the first detector of its kind to employ event-mode data acquisition, and is compatible with microsecond pixel transit times. The detector geometry enables optimizes experimental efficiency (*i.e.* elemental fluorescence per incident X-ray) while also placing no limits on sample size or scan range.

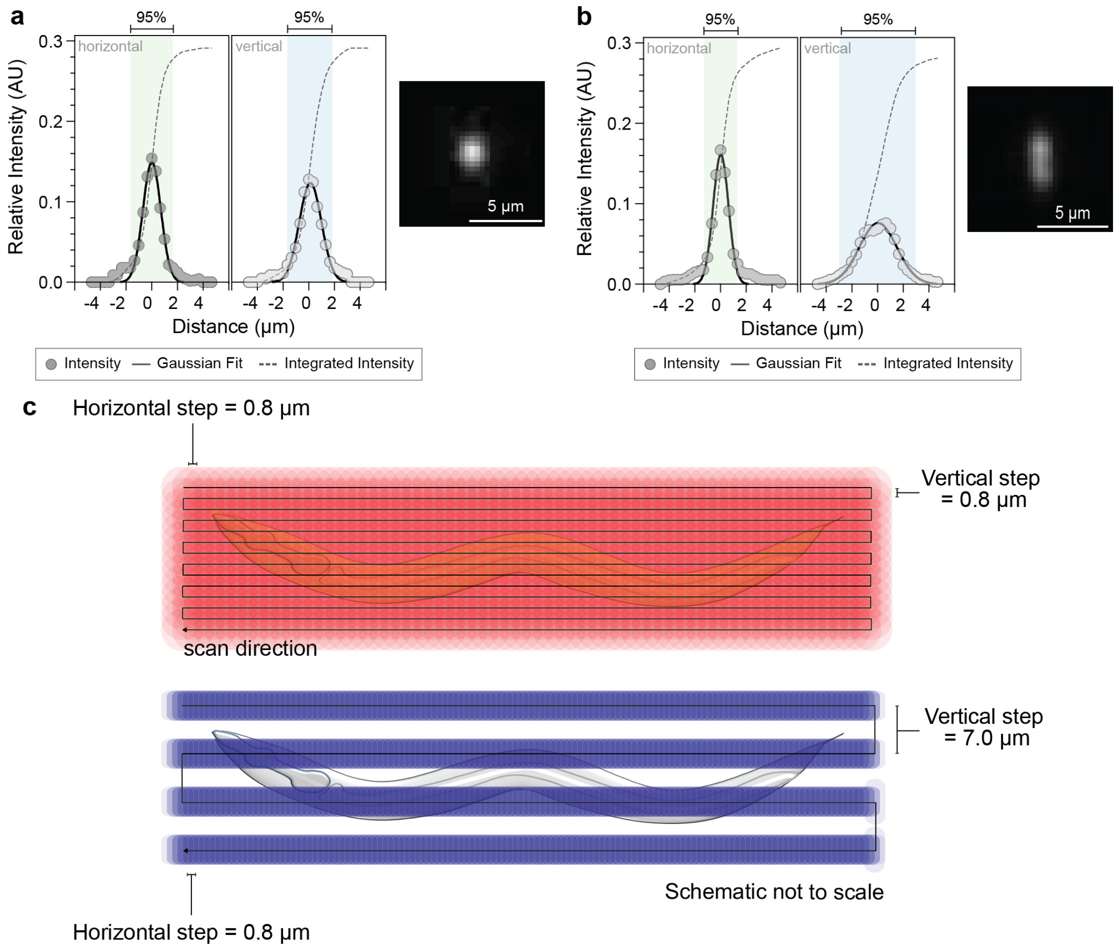

Supplementary Figure 1: X-ray beam profile and schematic of φXANES scanning approach. **(a)** For high dose φXANES a beam profile was used with a full width half max (FWHM) of a Gaussian distribution fitted to the beam profile with the spatial extent of illumination of 2.1 µm in horizontal (*x*-axis) and 2.8 µm in the vertical (*y*-axis). The beam was continuously scanned across the sample and resultant XRF spectra were binned at 0.8 µm intervals. The sample was also stepped 0.8 µm in the vertical, resulting in overlapping fields of illumination for each horizontal transits. Maps produced using this high dose protocol had a pixel area of 0.64 μm^2 and total pixel numbers in excess of 800,000 per map with a pixel transit time of 15.6 msec. This oversampling produced higher definition images with high sensitivity but exposed the sample to significantly greater incident radiation than the low dose method. **(b)** The low dose φXANES used an asymmetrical beam profile, FWHM 1.5 µm in *x* and 3.8 µm in *y*. Spectra were again binned at 0.8 µm in the *x*-direction, though stepping in the *y*-axis was increased to 7 µm. Resulting maps contained ~100,000 pixels of 5.6 μm^2 area with sample exposed to a significantly reduced dose,

predominantly through a reduced pixel transit time of 1.9 msec. c) Schematic representation of these two scanning parameters (not to scale).

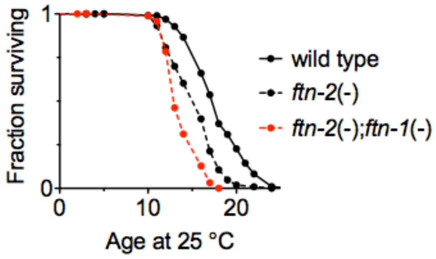

Supplementary Figure 2: Loss of all ferritin reduces median lifespan. Kaplan-Meier survival curve showing *ftn-2*(*ok404*); *ftn-1*(*ok3625*) null mutants have significantly reduced longevity compared to wild type ($p < 0.001$). Median life span at 25 °C: wild type 18 days, $n = 97$; *ftn-2*(*ok404*) 16 days, $n = 103$; and *ftn-2*(*ok404*); *ftn-1*(*ok3625*) 13 days, $n = 93$.

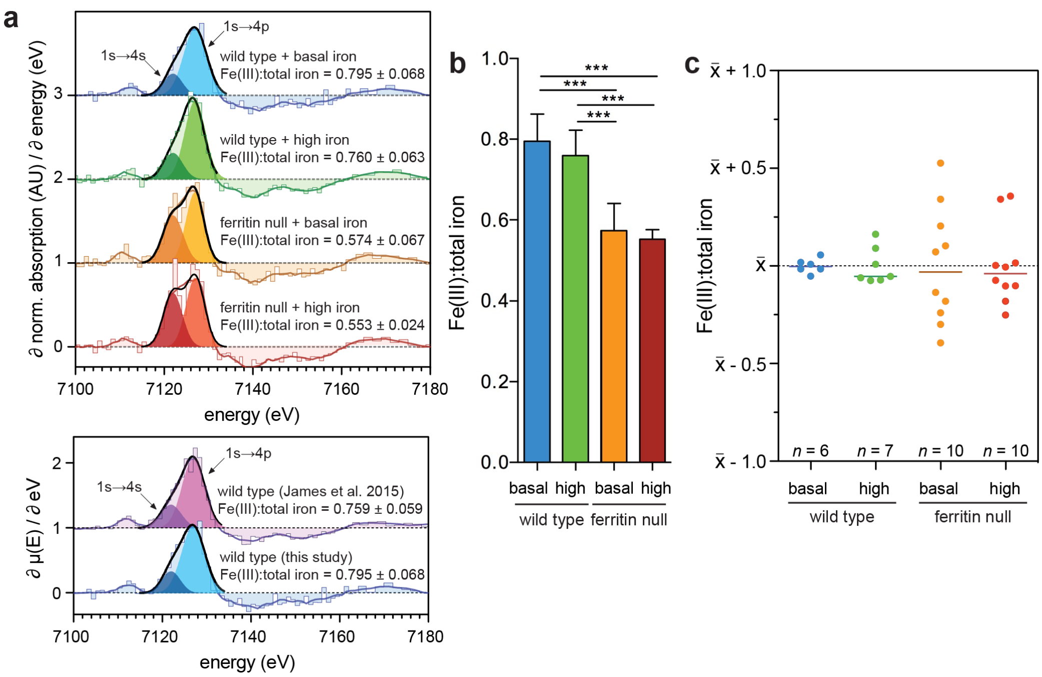

Supplementary Figure 3: Loss of ferritin increases variation in Fe(III):total iron ratio between individuals. **(a)** First derivative iron XANES spectra shown as raw (colored bars) and smoothed (3 point boxcar; colored lines) data corresponding to XANES shown in **Fig. 2c**, and were modelled as the sum of two Gaussians. The intensity associated with the 1s → 4s (dark shaded curves) and 1s → 4p (light shaded curves) transitions reflect the Fe(III):total iron (reported ± 95% confidence interval). The first derivative iron XANES spectra for all pixels and derived Fe(III): total iron ratio collected in this study (blue, derived from n = 3, where n is number of specimens) did not differ from previous XANES measurements of equivalent wild types on basal iron (purple, n = 3)[3]. For all subsequent analyses these two data sets were pooled. **(b)** This Fe(III):total iron varied significantly between genotypes and iron treatment (one way ANOVA with Tukey's *post hoc* test; *** $p < 0.001$). **(c)** Loss of ferritin significantly increased variability in the ability to regulate relative abundance of Fe(II) between individual animals (Bartlett's test for homogeneity of variances $\chi^2 = 16.54$; $p < 0.001$). As data on equivalent anatomical regions from each animal were not acquired, some individuals were excluded from this analysis due to low iron content (poor counting statistics) in the scanned region. The mean centred data for individual Fe(III):total iron is presented with a horizontal bar representing the media.

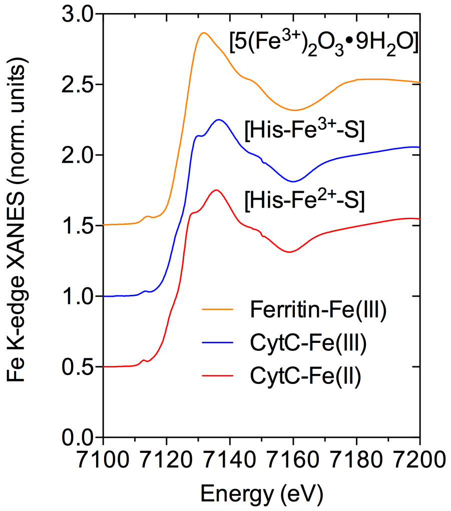

Supplementary Figure 4: Iron XANES spectra from reduced and oxidised cytochrome *c* (horse heart, Sigma) and ferritin (horse spleen, Sigma). Protein standards were used without further purification. For ferritin and oxidised cytochrome *c*, protein was dissolved in phosphate buffer (0.1 M, pH 7.0) to give a final concentration of 5 mM. Reduced Cytochrome *c* was prepared similarly, but the protein was reduced using a 6-fold excess of $Na_2S_2O_4$ under anerobic conditions (N_2 atmosphere). All standards were diluted to 2.5 mM via addition of 30% (v/v) glycerol and stored in liquid-N_2 until measured. Iron K-edge X-ray absorption spectra were recorded at the Australian Synchrotron XAS beamline using a Si(111) double-crystal monochromator. A Kirkpatrick-Baez mirror pair focused the beam to 0.2 mm (vertical) × 0.3 mm (horizontal), and the X-ray fluorescence was detected with a 100 element germanium fluorescence detector (Canberra). Data were collected from four separate scans and to avoid damage from excess X-ray exposure the samples were moved 200 μm vertically between collections. To monitor potential photodecomposition individual scans were energy-calibrated and compared using the program AVERAGE (from the Australian National Beamline Facility project). Scans were performed at 10 K using a closed-cycle helium cryostat. All spectra were energy-calibrated using an iron foil standard, with 7112 eV assigned as the first inflection point. XANES spectra had background subtraction and normalization methods implemented by ATHENA, an interface to IFEFFIT. For clarity the integrated XANES spectra for each compound have been offset vertically.

Supplementary Table 1: Proportion of pixels unique for each MANTiS identified region of interest (ROI) from φXANES.

		Pixels per ROI						*Percentage of pixels per ROI in total scan area*						
**Sample**	***n* (pixels per worm)**	**ROI1**	**ROI2**	**ROI3**	**ROI4**	**ROI5**	**ROI6**	**ROI1**	**ROI2**	**ROI3**	**ROI4**	**ROI5**	**ROI6**	**excluded**
**Wild type + high iron**														
**1**	65902	9738	7285	12592	10831	334	7441	14.78	11.05	19.11	16.44	0.51	11.29	26.83
**2**	74299	12736	2469	15409	8768	503	12790	17.14	3.32	20.74	11.80	0.68	17.21	29.10
**3**	68102	9730	1966	15605	8098	302	8624	14.29	2.89	22.91	11.89	0.44	12.66	34.91
**4**	48501	4900	820	13618	5672	64	6063	10.10	1.69	28.08	11.69	0.13	12.50	35.80
**5**	54667	9945	5569	8722	9222	482	7074	18.19	10.19	15.95	16.87	0.88	12.94	24.97
**6**	69813	9817	4803	22647	6187	127	7387	14.06	6.88	32.44	8.86	0.18	10.58	26.99
**7**	29582	2212	182	5017	5638	48	5988	7.48	0.62	16.96	19.06	0.16	20.24	35.48
Percentage of total measured pixels								**13.72**	**5.23**	**22.31**	**13.80**	**0.43**	**13.92**	**30.59**
Percentage of pixels in each ROIs for all worm pixels (i.e. excluding background)								**19.77**	**7.54**	**32.15**	**19.88**	**0.61**	**20.05**	
**ferritin null + high iron**														
**1**	31826	64	153	5891	12634	1537	6279	0.20	0.48	18.51	39.70	4.83	19.73	16.55
**2**	41547	126	340	5757	16206	1911	10327	0.30	0.82	13.86	39.01	4.60	24.86	16.56
**3**	63642	231	456	8172	23575	5367	13465	0.36	0.72	12.84	37.04	8.43	21.16	19.45
**4**	59603	267	2090	7258	18335	7924	11918	0.45	3.51	12.18	30.76	13.29	20.00	19.82
**5**	71421	174	2028	10617	24350	6102	12324	0.24	2.84	14.87	34.09	8.54	17.26	22.16
**6**	65554	137	73	11025	27819	2480	11546	0.21	0.11	16.82	42.44	3.78	17.61	19.03
**7**	75035	308	3556	10203	23620	7245	13203	0.41	4.74	13.60	31.48	9.66	17.60	22.52
**8**	59382	326	2133	5221	16599	7629	13775	0.55	3.59	8.79	27.95	12.85	23.20	23.07
**9**	22925	65	362	3230	5837	1053	5170	0.28	1.58	14.09	25.46	4.59	22.55	31.44
**10**	19262	19	86	4190	4003	844	3264	0.10	0.45	21.75	20.78	4.38	16.95	35.59
**11**	21676	53	496	1500	6484	1888	6117	0.24	2.29	6.92	29.91	8.71	28.22	23.70
**12**	17299	63	563	610	5785	2106	4840	0.36	3.25	3.53	33.44	12.17	27.98	19.26
**13**	10157	43	8	546	3853	649	2536	0.42	0.08	5.38	37.93	6.39	24.97	24.83
Percentage of total measured pixels								**0.32**	**1.88**	**12.55**	**33.08**	**7.86**	**21.70**	**22.61**
Percentage of pixels in each ROIs for all worm pixels (i.e. excluding background)								**0.41**	**2.43**	**16.21**	**42.74**	**10.16**	**28.04**	
Cumulative Fe(III):total iron								***0.91±0.09***	***0.87±0.09***	***0.74±0.21***	***0.68±0.09***	***0.68±0.06***	***0.68±0.08***	

References:

1. Ryan, C.G. *et al.* Maia X-ray fluorescence imaging: Capturing detail in complex natural samples. *JPCS* **499**, 012002 (2014).

2. Kirkham, R. *et al.* The Maia Spectroscopy Detector System: Engineering for Integrated Pulse Capture, Low‐Latency Scanning and Real‐Time Processing. *AIP Conf. Proc.* **1234**, 240-243 (2010).

3. James, S.A. *et al.* Direct in vivo imaging of ferrous iron dyshomeostasis in ageing Caenorhabditis elegans. *Chem. Sci.* **6**, 2952-2962 (2015).
